# Artificial intelligence to unlock real‐world evidence in clinical oncology: A primer on recent advances

**DOI:** 10.1002/cam4.7253

**Published:** 2024-06-20

**Authors:** Alex K. Bryant, Rafael Zamora‐Resendiz, Xin Dai, Destinee Morrow, Yuewei Lin, Kassidy M. Jungles, James M. Rae, Akshay Tate, Ashley N. Pearson, Ralph Jiang, Lars Fritsche, Theodore S. Lawrence, Weiping Zou, Matthew Schipper, Nithya Ramnath, Shinjae Yoo, Silvia Crivelli, Michael D. Green

**Affiliations:** ^1^ Department of Radiation Oncology University of Michigan School of Medicine Ann Arbor Michigan USA; ^2^ Department of Radiation Oncology, Veterans Affairs Ann Arbor Healthcare System Ann Arbor Michigan USA; ^3^ Applied Mathematics and Computational Research Division, Lawrence Berkeley National Laboratory Berkeley California USA; ^4^ Computational Science Initiative, Brookhaven National Laboratory Upton New York USA; ^5^ Department of Pharmacology University of Michigan School of Medicine Ann Arbor Michigan USA; ^6^ Department of Internal Medicine University of Michigan School of Medicine Ann Arbor Michigan USA; ^7^ Department of Statistics University of Michigan Ann Arbor Michigan USA; ^8^ Center of Excellence for Cancer Immunology and Immunotherapy University of Michigan Rogel Cancer Center Ann Arbor Michigan USA; ^9^ Department of Pathology University of Michigan Ann Arbor Michigan USA; ^10^ Graduate Program in Immunology University of Michigan Ann Arbor Michigan USA; ^11^ Division of Hematology Oncology, Department of Medicine University of Michigan School of Medicine Ann Arbor Michigan USA; ^12^ Division of Hematology Oncology, Department of Medicine Veterans Affairs Ann Arbor Healthcare System Ann Arbor Michigan USA; ^13^ Graduate Program in Cancer Biology University of Michigan Ann Arbor Michigan USA; ^14^ Department of Microbiology and Immunology University of Michigan School of Medicine Ann Arbor Michigan USA

**Keywords:** Artificial Intelligence, Cancer Outcomes Research, Large language models, Observational Data, prognostic factor

## Abstract

**Purpose:**

Real world evidence is crucial to understanding the diffusion of new oncologic therapies, monitoring cancer outcomes, and detecting unexpected toxicities. In practice, real world evidence is challenging to collect rapidly and comprehensively, often requiring expensive and time‐consuming manual case‐finding and annotation of clinical text. In this Review, we summarise recent developments in the use of artificial intelligence to collect and analyze real world evidence in oncology.

**Methods:**

We performed a narrative review of the major current trends and recent literature in artificial intelligence applications in oncology.

**Results:**

Artificial intelligence (AI) approaches are increasingly used to efficiently phenotype patients and tumors at large scale. These tools also may provide novel biological insights and improve risk prediction through multimodal integration of radiographic, pathological, and genomic datasets. Custom language processing pipelines and large language models hold great promise for clinical prediction and phenotyping.

**Conclusions:**

Despite rapid advances, continued progress in computation, generalizability, interpretability, and reliability as well as prospective validation are needed to integrate AI approaches into routine clinical care and real‐time monitoring of novel therapies.

## Graphical abstract

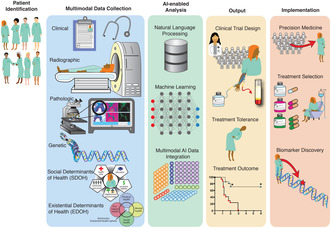

## 1 INTRODUCTION

**FIGURE 1:**
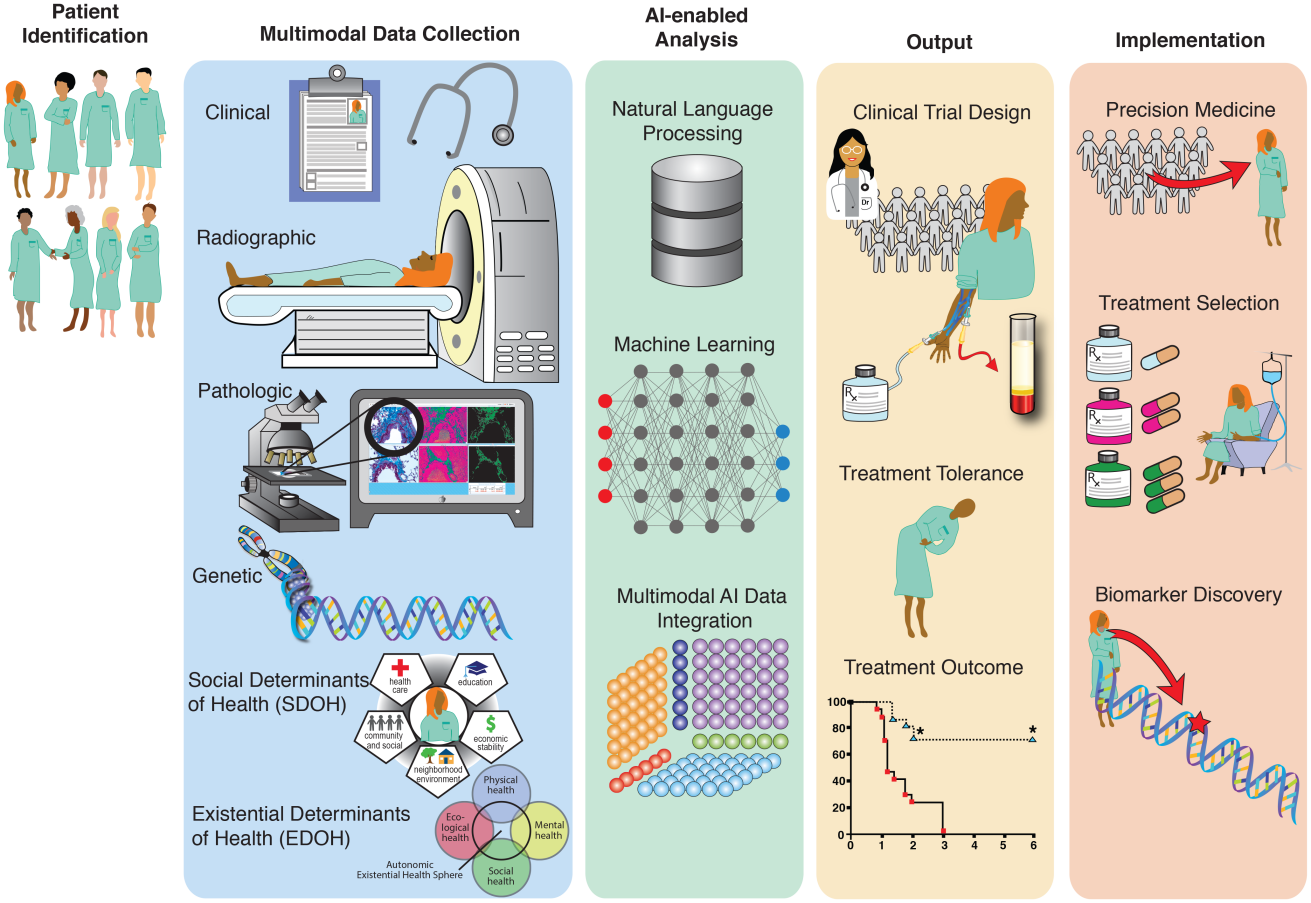
Ongoing and Potential Integrations of AI in Oncology. There is increasing efforts to combine clinicopathologic factors, social and existential determinants of health, and radiographic features using AI approaches to evaluate cancer therapy treatment outcomes and improve cancer care.

The daily practice of oncology is filled with uncertainty: the unclear real‐world effectiveness of novel therapies; the lack of precise prognostic information relevant to individual patients; a plethora of management decisions for which there is no prospective evidence; and unknown real‐world treatment toxicity rates. These gaps have increased the value of real world evidence that rigorously defines patient prognosis, treatment response, and toxicity outside of clinical trials, and of risk prediction models that can reduce diagnostic and prognostic uncertainty.^1^, ^2^ However, much of the narrative experience of oncology exists within unstructured medical records such as oncologist notes, discharge summaries, and radiographic or pathologic reports. Critical prognostic information is also embedded within complex multimodal datasets such as radiographic images, tumor and germline sequencing, germline sequencing, and histology slides. The significant effort required for annotation, curation, and interpretation of these unstructured data sources prevents healthcare systems from rapidly learning from emerging experiences with novel therapies.^3^ There is a critical need for flexible approaches that capture and distill the complexities of real‐world cancer care. Artificial intelligence (AI), which refers to the use of complex computer algorithms to provide solutions and inform decision making for unsolved problems, has shown significant potential in this area. Neural network‐based AI approaches such as natural language processing (NLP) and deep learning (DL) are revolutionizing the collection and interpretation of real‐world oncological data and are beginning to touch many aspects of cancer care.^4^, ^5^ In this review, we highlight recent advances in NLP, DL, and other AI approaches being applied to routinely collected oncological data to advance our understanding of real‐world cancer care and discover novel therapies (Figure 1).

## 2 AI APPLICATIONS IN CANCER SCREENING, DIAGNOSIS, AND STAGING

Prospective trials have highlighted the benefits of screening for breast, colorectal, lung, cervical, and prostate cancer,^6^, ^7^, ^8^ but there is continuing controversy over which real‐world patient subgroups benefit. There is also controversy whether the small absolute survival benefits seen in many screening trials is outweighed by the increased detection of indolent disease and unnecessary workup leading to emotional and financial stresses.^9^ As a result, uptake of some screening procedures remain low; for example, <15% of eligible patients undergo low‐dose computed tomography screening for lung cancer^10^ and prostate‐specific antigen screening rates have declined in recent years due in part to concerns of overdiagnosis of indolent cancers.^11^, ^12^ Given these challenges, there is increasing interest in applying AI approaches to augment the interpretation of screening studies and maximize the potential benefits of screening while minimizing harms of overdiagnosis.

Screening approaches include direct visualization (cutaneous malignancies), radiographic evaluations (breast and lung malignancies), endoscopy (gastrointestinal malignancies), blood markers (prostate cancer), and tissue sampling (cervical cancer). Advances in image analysis have enabled DL approaches to contribute to the interpretation of most of these screening modalities. Convolutional neural networks (CNNs) use a grid‐like topology using layers of filters or “convolutions” to model high dimensional, non‐linear feature spaces such as those found in medical imaging datasets. CNNs have shown impressive accuracy in multiple studies for skin cancer detection,^13^, ^14^ in some cases outperforming human raters^15^, ^16^ with positive predictive values for melanoma in the 0.80–0.90 range and negative predictive values in the 0.90–0.95 range.^17^ There are significant challenges in applying these algorithms in routine care, where confounding visual features such as artifact from pen markings and crusted lesions can cause a substantial decrement in model accuracy.^18^ In colorectal cancer screening, CNNs have been shown in a randomized controlled trial to decrease the endoscopic miss rate of adenomas by half, primarily by increasing the detection of flat, small, and subtle neoplastic lesions.^19^ Moreover, CNNs also show promise in improving the cost‐effectiveness of screening^20^ and in mitigating quality disparities.^21^ CNNs can improve the efficiency and accuracy of cervical cytology, with one study showing higher specificity compared to conventional screening and lower rates of referral for colposcopy;^22^ such approaches may be especially relevant in resource‐limited settings where cervical cancer incidence is highest.^23^ While AI‐assisted mammography interpretation holds promise for breast cancer detection,^24^ studies have yet to conclusively establish the benefit over expert radiographic review and large prospective validation studies are needed.^25^ In lung cancer, a three‐dimensional CNN termed Sybil has shown impressive accuracy in detecting short‐term risk of lung cancer from a single low‐dose computed tomography scan of the chest, with a 1‐year area under the receiver operating characteristic curve of 0.86–0.94 in validation sets.^26^ Sybil and similar models trained on computed tomography data may refine risk prediction in lung cancer screening and reduce false‐positive rates seen in screening trials,^6^ particularly when combined with longitudinal clinical data and when incorporating repeated screens. A critical weakness of many imaging‐based AI algorithms is the narrowness of training datasets, which are often drawn from European‐centric patient populations, single medical centers, and a limited set of radiological scanner vendors, each of which can produce biased, poorly calibrated models which fail to generalize. As such, multi‐institutional validation studies are needed to test models in the full range of scanning technologies and patient populations reflective of routine clinical care.

Many groups have studied the application of AI techniques to aid interpretation of digitized pathology slides of tumor biopsies and cytology.^27^ The recent availability of whole slide imaging systems has produced a flood of histopathological imaging data which is increasingly used to train DL models.^28^ Major tasks include tumor segmentation and classification,^29^, ^30^ automated characterization of the tumor microenvironment (such as tumor‐infiltrating lymphocyte density, which may predict responsiveness to immunotherapies),^31^ detection of metastases in tumor draining lymph nodes,^32^ and prediction of tumor mutational status,^33^, ^34^, ^35^ among many others.^27^ Serious challenges remain in ensuring model stability across disparate laboratories and preparation techniques, managing the massive quantity of imaging data produced by whole‐slide scanners, and defining the clinical utility and cost‐effectiveness of digitization efforts and AI model deployment. Clinical questions also remain about the treatment implications of highly sensitive AI algorithms. For example, deployment of a model that significantly increases detection of micro metastases may expose patients to intensified therapy and over‐treatment without a clearly defined clinical benefit. Carefully controlled prospective studies are therefore needed to assess the patient‐centered benefit of incorporating model outputs into cancer staging and management decisions.

Accurate interpretation of radiographic images is critical in assigning clinical stages to cancer patients, which largely dictates subsequent treatments. DL models are rapidly being applied to baseline staging images to provide more accurate staging and risk stratification. DL models have been applied in breast cancer to improve detection of subclinical axillary nodal metastases on ultrasound,^36^ in head and neck cancer to predict the presence of extra nodal extension in pre‐treatment computed tomography scans,^37^ and in lung cancer to predict pathologic node positivity in the mediastinum,^38^ among others.^39^ While these tools can reduce the burden of labor‐intensive cognitive tasks and improve diagnostic accuracy, it is unknown whether allowing clinical management to be influenced by the probabilistic outputs of DL models will improve patient‐centered outcomes like quality of life or survival, and prospective studies are needed to more clearly define the benefits of these novel algorithms.

## 3 AUTOMATED EXTRACTION OF PATIENT AND CANCER CHARACTERISTICS

Once a cancer is appropriately screened, diagnosed, and staged, this critical information is typically transcribed in free‐text notes from oncologists or other front‐line providers. Additional crucial data such as performance status, symptoms from medical comorbidities, specific pathological and radiological findings, medication compliance, and family history are also primarily contained in free‐text form. While structured clinical data derived from electronic medical records—such as laboratory results, diagnosis codes, and procedures—provide important prognostic information, much of the crucial patient and cancer features that dictate subsequent treatment are locked in unstructured text and invisible to traditional methods of analysis that require structured clinical data. As such, there have been major efforts to develop NLP approaches—both deterministic rule‐based systems and probabilistic systems based on large language models (LLMs)—to facilitate analysis of free text and enable real‐time case identification, patient profiling, and disease characterization.

Deterministic rule‐based NLP systems have demonstrated excellent performance in the extraction of targeted features from unstructured text in scenarios where a limited lexicon is used to describe the features of interest. The Leo framework has been applied successfully to extract pathologic stage from surgical pathology reports^40^ and the CLAMP (Clinical Language Annotation, Modeling, and Processing) framework for extraction of tumor size, stage, and biomarker results.^41^ Custom NLP pipelines engineered for extraction of specific features can include initial development of a lexicon reflecting the features of interest, followed by term expansion using computational methods such as word embeddings derived from statistical language models. Free‐text extracted around the term list is then processed using standardized ontologies and rule‐based NLP tools to produce the final feature vector. This process is exemplified in a recent report describing the development of a high‐performance algorithm to extract new diagnoses of metastatic prostate cancer in the Veterans Affairs system based on a complex logic of anatomical and diagnostic text patterns.^42^ Such pipelines require substantial development time and may be applicable only to the healthcare setting in which they were developed, with idiosyncratic note types, data structures, and vocabularies. There have therefore been efforts to develop generalizable NLP algorithms that use rule‐based systems like cTAKES to map concepts in unstructured free text to large cancer ontologies.^43^ While promising, the results of these generalized systems have not been extensively validated and may not produce reliable outputs for all features.

A fundamental weakness of rule‐based NLP systems is a lack of flexibility and context‐dependence, weaknesses that may be overcome in the era of transformer‐based LLM like GPT,^44^ LLAMA,^45^ and Claude^46^ that model semantic context. While the training of massive language models has been limited by the dearth of large, diverse corpora of deidentified clinical text for training, early efforts have shown remarkable results in feature extraction even in clinically ambiguous contexts.^47^, ^48^ LLMs may quickly subsume previous rule‐based NLP systems as a “one size fits all” solution for feature extraction, document classification, and other traditional clinical NLP tasks.^48^ Importantly, the optimal method of extracting structured variables from large language models is not yet clear, and the emerging field of prompt engineering has highlighted the sensitivity of generative LLM outputs to the precise wording and sequencing of prompts.^47^, ^49^ While the application of these models is still in its infancy, the highly visible success of OpenAI's ChatGPT is producing a massive acceleration of effort in this area and LLMs will find immediate application in feature extraction tasks across medicine.

## 4 DL FOR OUTCOME AND TOXICITY PREDICTION

Cancer researchers have made great strides in unraveling the major factors affecting long‐term prognosis. These include a refined understanding of cancer staging and patterns of spread,^50^ competing mortality prediction,^51^ molecular tumor subtyping,^52^ and heterogenous treatment effects,^53^ in addition to the many novel therapies and treatment approaches entering routine practice each year. While our ability to use these factors to better predict outcomes of subgroups has improved dramatically, the clinical course for individual patients has remained stubbornly unpredictable. There is a need for new approaches to dynamically predict treatment efficacy, toxicity, and non‐cancer mortality events throughout the patient's clinical course, enabling early interventions for supportive care and better personalizing treatment selection. The confluence of increasing centralization of multimodal clinical data and integrative AI approaches can bring the field closer to realizing the dream of personalized medicine.

The advancing flood of multimodal data—radiographic, genomic, pathomic, and clinical—has produced a flowering of AI approaches to integrate these fundamentally disparate data sources and identify novel connections across modalities. In rectal cancer, radiomic features of the pre‐treatment pelvic MRI were combined with pathomic features from tumor biopsies to improve prediction of pathological complete response after neoadjuvant therapy, outperforming radiomic or pathomic features alone.^54^ In breast cancer, whole‐slide pathomic images and clinical variables were integrated to predict response to neoadjuvant chemotherapy in triple negative breast cancer through federated learning.^55^ This is a novel example of edge computing which does not require central pooling of data for model training and therefore protects data privacy while improving feasibility.^55^ In lung cancer, radiomic analysis of computerized tomography (CT) images could predict lung cancer EGFR genotype non‐invasively with area under the curve (AUC) ranging from 0.75 to 0.81,^56^ and integration of radiomic, pathomic, and genomic features improved prediction of immunotherapy responses beyond unimodal measures alone.^57^ Similar results have been found in serous ovarian cancer^58^ and prostate cancer^59^ among many others.^60^ The optimal method of multimodal data fusion continues to evolve and can range from early fusion, in which raw features from each modality are fed into a single neural network with minimal pre‐processing, to late fusion, in which separate networks are trained on each modality and then aggregated for a combined prediction.^60^ Interpretability of these incredibly complex networks remains a challenge, though attentional heat maps can assist human observers in identifying the most salient features and produce new avenues for mechanistic research.^60^, ^61^

While many model development efforts have focused on using baseline clinical data for prediction, incorporating longitudinal data could increase predictive accuracy and relevance of model predictions to on‐the‐fly clinical decision making. Bayesian machine learning (ML) methods have been developed and applied to patients with diffuse large B‐cell lymphoma, incorporating baseline clinical measures and longitudinal biomarker measurements to dynamically update disease progression and survival risks.^62^ Flexible longitudinal ML approaches are increasingly applied in other domains including Bayesian models for prediction of renal survival after kidney transplant using longitudinal laboratory values^63^ and recurrent neural networks to predict acute kidney injury in the Veterans Affairs system, incorporating high‐dimensional clinical text features.^64^ DL approaches have been applied to infer formal tumor response criteria from radiology text reports^65^, ^66^ as well as approaches like term frequency inverse documentation weighting and support vector machines, which is a supervised learning model that uses classification and regression analysis to define optimal hyperplanes for data discrimination.^67^ Groups have also developed medical concept extraction approaches to infer therapeutic benefit from other sources including operative reports, pathology reports, and the narrative medical record.^68^

LLMs trained on clinical text have also been proposed as general‐purpose prediction engines that can quickly produce best‐in‐class risk predictions for virtually any clinical outcome.^69^ Well‐trained LLMs appear to produce large improvements in prediction accuracy over traditional ML approaches using structured clinical features (such as diagnosis codes and laboratory values) alone.^69^ Important questions remain about the optimal model size, the need for a single foundational model versus multiple smaller models fine‐tuned for distinct prediction tasks, model architecture, pre‐training method, and need for site‐specific fine‐tuning of model parameters at individual hospitals in a network. Operationalized LLMs will also likely require periodic re‐tuning and vocabulary expansions to accommodate shifts in medical terminology usage and new drug names or procedures. Finally, LLM prediction models will likely benefit from incorporation of orthogonal datasets not directly present in the electronic medical record, such as three‐dimensional radiographic images and genomic sequencing data. The optimal architecture for incorporating LLM predictions with other multimodal datasets remains unknown.

Even successful cancer therapies can have lifelong toxicities, some of which can be functionally devastating. Unfortunately, many acute and long‐term toxicities therapies remain largely unpredictable despite our increasing reliance on genomically‐tailored therapies, immunotherapy, and precision radiotherapy. This problem is likely more acute in real‐world settings compared to clinical trials, as patients enrolled on clinical trials are well known to have more extensive support networks and fewer medical comorbidities, which can improve treatment tolerance.^70^ AI approaches are increasingly used to fill the knowledge gap by describing real‐world, longitudinal patient experiences, such as capturing pain, fatigue, and other patient‐reported side effects from the medical record.^71^, ^72^, ^73^ A challenge with many NLP approaches is the need to generate a lexicon of terms for a given toxicity; however, the use of weak labeling and large standardized ontologies may help overcome this challenge.^74^, ^75^ Cancer patients can discontinue therapy due to symptom burden in a specific domain as well as the overall perception of diminished quality of life, and traditional NLP models have shown success in defining the clinical rationale for treatment discontinuation.^76^ NLP monitoring of Twitter can even provide insight into real‐world drug tolerance.^77^, ^78^ These studies highlight that AI approaches may be able to monitor a variety of data sources to enable early warning of clinicians when patients are developing toxicities as well as aid in post‐approval surveillance for cancer treatments.

## 5 AI INTEGRATION INTO ROUTINE PRACTICE

There are emerging performance frameworks developed with the Food and Drug Administration (FDA) for implementation of AI as a medical device,^79^ and AI is increasingly integrated into routine workflows in multiple specialties such as aiding radiologist interpretation and assisting in adaptive radiotherapy planning.^80^ AI systems can even generate preliminary treatment recommendations, though these remain rudimentary and have yet to inform routine clinical practice.^81^ While the burgeoning AI literature is replete with model‐building exercises and impressive performance on test datasets, prospective validation of prediction algorithms in randomized controlled trials has been scarce. One recent trial in radiation oncology showed that an ML model predicting unplanned ED visits or hospitalizations could help direct intensive clinical monitoring during radiation and reduce acute care visits.^82^ Importantly, this trial did not randomize patients to ML‐directed care versus usual care, but rather used ML to identify patients at risk of acute care visits who were then randomized to usual care versus intensive monitoring. Another recent trial used a stepped‐wedge design to evaluate the impact of an ML algorithm to identify patients at high risk of death within 6 months, whose providers were then prompted to consider initiating a serious illness conversation with the patient.^83^ This strategy led to an increase in serious illness conversations and reduction in end‐of‐life systemic therapy administration.^83^ ML tools have been developed to identify patients eligible for enrollment on clinical trials of new cancer therapies^84^, ^85^ and to explore inefficiencies in restrictive clinical trial enrollment criteria relative to real‐world populations.^86^ Despite these efforts, prospective validation of ML models and rigorous testing of their impact on clinical care remain disappointingly rare.

Recent advances in DL have significantly impacted radiation oncology, particularly in the automatic generation of organ‐at‐risk (OAR) and target contours on computed tomography radiation planning scans. These algorithms, primarily based on CNNs, have shown remarkable accuracy and efficiency in delineating OARs and tumor targets and have rapidly entered routine clinical practice.^87^ A noteworthy development is the integration of 3D CNNs, which better capture the spatial relationships in volumetric data, leading to improved contour accuracy compared to traditional approaches.^88^ Generative adversarial networks (GANs) have been employed to augment training datasets and to perform data normalization, enhancing model robustness across different imaging modalities and protocols.^89^ AI applications in radiation treatment planning have also advanced notably. These models expedite the treatment planning process by automating dose distribution predictions and beam angle and intensity selections, tasks that are traditionally manual and time‐consuming.^90^ However, challenges such as ensuring model generalizability across different patient demographics and smooth integration into existing clinical workflows persist.

Technical challenges in training and validating complex ML models can also hinder performance and deployment efforts. ML models generally require many thousands of training examples to perform adequately, and performance should then be tested on large, preferably external validation datasets derived from an entirely different population than the training data to prove generalizability.^91^ Well‐powered training data and external validation datasets may not be practically available for some prediction problems. Further, while external validation has typically been held as the gold‐standard in testing model performance, future models may be increasing trained and deployed in only one hospital or healthcare system,^69^ suggesting that rigorous, repeated internal validation may be more important than external generalizability.^92^ Some argue that there is no such thing as a truly validated prediction model due to the constant performance drift over time and practice settings that requires periodic model updating.^93^ Technical aspects of the model training process can also affect performance and generalizability such as choosing among multiple architectures, tuning hyper parameters, and guarding against overfitting with methods such as cross‐validation. AI implementation efforts need well‐designed governance structures to provide oversight on these issues while also being flexible enough to rapidly identify, test, and deploy technical advancements in this fast‐moving field.

A core challenge to implementing models remains physician and patient trust in model outputs. Both physicians and patients have interests in understanding the underlying principles of clinically deployed AI algorithms.^94^, ^95^ While approaches like Local Interpretable Model‐Agnostic Explanations (LIME),^96^ Shapley additive explanation (SHAP),^97^ and many others^98^ provide frameworks for explainable AI,^99^ there remain deep limitations to these approaches.^100^ Further undermining trust is the well‐documented biases apparent in models that have not been trained on appropriately diverse data sources, including racial biases.^101^ Equity must be a conscious design principle and training on diverse patient populations should be a requirement before model deployment. Finally, clinical care requires robust models that can be applied in diverse healthcare environments and which are temporally stable and continuously monitored.^102^ Thus, advances in reliability, stability, portability, adaptability, and equity are needed as AI continues to integrate into oncology practice.

## 6 CONCLUSION

Cancer impacts more than 20 million new individuals each year. Understanding and integrating the totality of cancer experiences is needed to make improvements in cancer care, but the unstructured nature of the medical record has made large‐scale data integration highly resource intensive under classical methods. AI approaches are filling this gap and have already revolutionized the collection, analysis, and interpretation of routinely collected unstructured health data. This includes tools aiding in patient selection for cancer screening, interpreting radiographic studies, accurately staging new cancer patients, profiling patients using unstructured medical text, predicting treatment response and toxicity, generating novel connections between disparate data modalities, and directing clinical trial enrollment, among others. While there is great excitement surrounding these tools, their integration into routine care has been hampered by a relative lack of high‐quality prospective validation studies and randomized clinical trial to prove the benefit of AI‐assisted care. There are also ongoing concerns regarding the reliability, generalizability, accuracy, equity, and temporal stability of deployed prediction models. Despite the challenges, these methods hold incredible promise for improving the care of patients with cancer.

## AUTHOR CONTRIBUTIONS

**Alex K. Bryant:** Conceptualization (equal); writing – original draft (equal). **Rafael Zamora‐Resendiz:** Writing – original draft (equal). **Xin Dai:** Writing – original draft (equal). **Destinee Morrow:** Writing – original draft (equal). **Yuewei Lin:** Writing – original draft (equal). **Kassidy M. Jungles:** Writing – original draft (equal). **James M. Rae:** Writing – original draft (equal). **Akshay Tate:** Writing – original draft (equal). **Ashley N. Pearson:** Writing – original draft (equal). **Ralph Jiang:** Writing – original draft (equal). **Lars Fritsche:** Writing – original draft (equal). **Theodore S. Lawrence:** Writing – original draft (equal). **Weiping Zou:** Writing – original draft (equal). **Matthew Schipper:** Writing – original draft (equal). **Nithya Ramnath:** Writing – original draft (equal). **Shinjae Yoo:** Funding acquisition (equal); writing – original draft (equal). **Silvia Crivelli:** Funding acquisition (equal); writing – original draft (equal). **Michael D. Green:** Conceptualization (equal); funding acquisition (equal); supervision (lead); writing – original draft (equal).

## FUNDING INFORMATION

Lung Precision Oncology Program (VA 150CU000182; PI Ramnath), LUNGevity (2021–07, PI Green), NCI (R01CA276217, PI Green), Veterans Affairs (I01 BX005267; PI Green), Melanoma Research Alliance (MRA 689853; PI Green), Veterans Affairs (MVP064; PI Green, Crivelli, Yoo).

## CONFLICT OF INTEREST STATEMENT

Authors report no conflicts of interest.

## Data Availability

No new data sets were developed as part of this manuscript.
